# Mild Biceps Tendonitis May Be Managed Nonoperatively During Shoulder Arthroscopy

**DOI:** 10.1016/j.asmr.2023.100785

**Published:** 2023-09-20

**Authors:** Jonathan J. Light, Wihan du Plessis, Matthew H. Adsit, Alexander T. Eckstrom, Amanda B. Firoved, Justin W. Griffin, Kevin F. Bonner

**Affiliations:** a Eastern Virginia Medical School, Norfolk, Virginia, U.S.A.; b Ohio Health-Riverside Methodist Hospital, Columbus, Ohio, U.S.A.; c Department of Orthopaedic Surgery, Virginia Commonwealth University, Richmond, Virginia, U.S.A.; d Jordan-Young Institute for Orthopedic Surgery & Sports Medicine, Virginia Beach, Virginia, U.S.A.

## Abstract

**Purpose:**

The purposes of this study were to determine whether patients with mild biceps tendonitis required revision surgery after the biceps tendon was not surgically treated, while addressing concomitant pathology, and to evaluate whether preoperative groove pain affected patient-reported outcomes.

**Methods:**

Patients who underwent shoulder arthroscopy between 2015 and 2018 by a single surgeon for rotator cuff pathology, debridement, and distal clavicular excision (DCE), with or without subacromial decompression (SAD), and where the biceps tendon was not surgically addressed were retrospectively identified. Inclusion criteria were mild LS (<50% hyperemic appearing biceps tendon arthroscopically), and a minimum 2-year follow-up. The primary outcome measure was the incidence of revision surgery. Secondary outcomes included American Shoulder and Elbow Surgeons (ASES) score, simple shoulder test (SST), pain level, and satisfaction scores. Two sample *t*-tests compared postoperative patient-reported outcomes based on the presence or absence of preoperative bicipital groove tenderness.

**Results:**

Sixty-four of 69 eligible subjects (93%) were evaluated at a minimum of 2 years postoperatively. One out of 64 subjects underwent revision to perform a biceps tenodesis. Overall, patients had high patient-reported outcome measures (PROMs) postoperatively. Ninety-seven percent of patients reported they would have the surgery again. The presence of preoperative bicipital groove tenderness had no effect on ASES (*P* = .62), SST (*P* = .83) scores, and postoperative pain (*P* = .65). Patients without bicipital groove pain had average respective ASES and SST scores of 93.70 ± 11.84 and 10.66 ± 2.47; those with bicipital groove pain averaged 92.00 ± 15.31 and 10.78 ± 1.87. There was no significant difference in overall satisfaction scores between patients with groove pain (9.42 ± 1.40) and those without (9.46 ± 1.38; *P* = .92).

**Conclusions:**

Patients with mild biceps tendonitis showed favorable outcomes with low revision rates and high patient satisfaction when the biceps tendon was not surgically addressed when the primary shoulder pathology was treated during arthroscopy, independent of preoperative groove pain.

**Level of Evidence:**

Level III, retrospective cohort study.

## Introduction

Tendinitis of the long head of the biceps brachii (LHB) tendon can be diagnosed arthroscopically by the presence of the "lipstick sign" (LS), as the tendon traverses within the bicipital groove over the anterosuperior humerus.^1^^,^^2^ The hyperemic appearance of the so-called LS results from inflammatory tenosynovitis.^2^ Although LHB tendinitis sometimes occurs in isolation, it is often present with a constellation of other shoulder pathologies, including rotator cuff tears or tendinosis, subacromial impingement, adhesive capsulitis, synovitis, labral tears, glenohumeral arthrosis, and acromioclavicular arthritis.^2^, ^3^, ^4^, ^5^, ^6^ Some estimate that around 90% of patients with LHB tendon pathology have concomitant rotator cuff tears, which lends support that the biceps tendon can be affected or often coexists with other shoulder pathology.^6^ LHB tendon pathology often needs to be addressed with concomitant pathology, such as rotator cuff tears. Additionally, there are varying degrees of tenosynovitis biceps tendon. However, it is not an uncommon scenario for the biceps pathology to be deemed mild but not normal when addressing other more substantial shoulder pathology. At what point should we pull the trigger and surgically address the biceps if it is not normal?

Persistent postoperative pain due to biceps tendinitis can lead to inferior outcomes or potential reoperation to address the biceps tendon, which is a concern for surgeons. This has led many surgeons to surgically address the biceps unless it looks completely normal. Two decades ago, many treatment algorithms favored addressing the biceps only for structural tears greater than 50%, or if unstable.^7^ What should our threshold be in the setting of just mild inflammation of the biceps, in the setting of what is felt to be a more significant concomitant shoulder pathology? It is not an uncommon intraoperative clinical dilemma to perseverate over one's threshold to perform a biceps tenodesis in the setting of mild pathology.

There is little argument that surgical intervention of the LHB tendon is indicated when severe tendinitis, structural tearing, or instability is present during shoulder arthroscopy.^5^^,^^8^ What is more controversial, and the focus of this study is whether intervention for a mild LS favors leaving the biceps alone, and, if left alone, does it adversely compromise the outcomes of the shoulder surgery? There are risks to tenodesis and tenotomy.^9^ Additionally, although postoperative rehabilitation may not be dramatically affected in the setting of concomitant rotator cuff repair and tenodesis, for other indications, tenodesis may make a recovery more onerous for patients who otherwise would not be in a sling for 4 to 6 weeks. Patients who underwent shoulder arthroscopy between 2015 and 2018 by a single surgeon for rotator cuff pathology, debridement, and distal clavicular excision (DCE), with or without subacromial decompression (SAD), and where the biceps tendon was not surgically addressed, were retrospectively identified. Inclusion criteria were mild LS (defined as a biceps tendon with less than 50% of its surface area that was hyperemic in appearance by arthroscopic examination), age between 18 and 89 years at the time of surgery, and a minimum 2-year follow-up. We also evaluated whether bicipital groove pain preoperatively affected patient-reported outcomes in this group. We hypothesized that avoiding LHB intervention in patients with mildly positive LS and addressing other concomitant pathologies felt to be the patient’s pain generator would result in favorable outcomes.

## Methods

**Fig 1:**
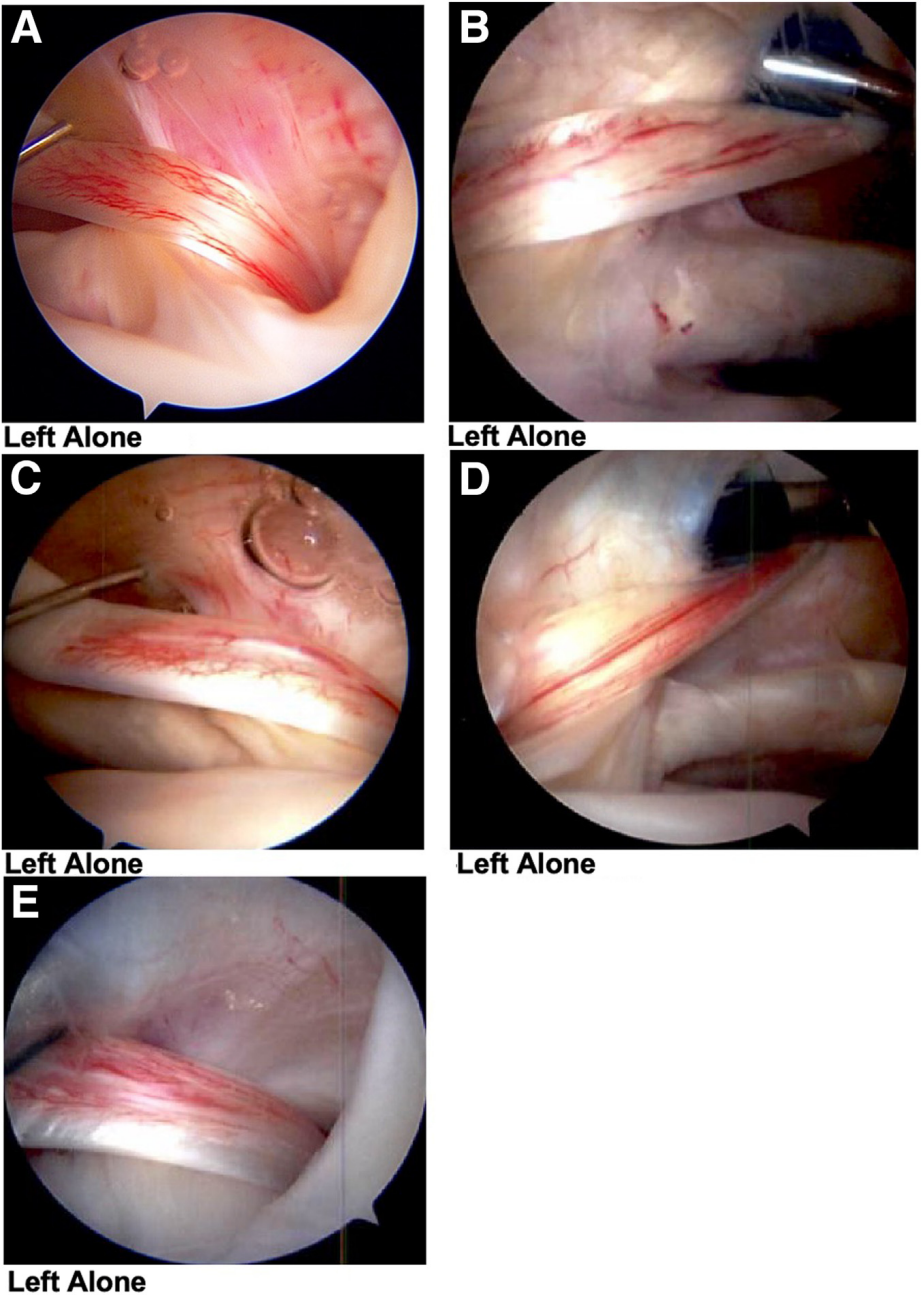
Examples A-E show posterior portal view arthroscopic images of patients from this study who exhibited mild to moderate biceps tendonitis “lipstick sign" during arthroscopy; these patients had no intervention performed on the biceps tendon. (A, C, and E are right shoulders, B and D are left).

**Fig 2:**
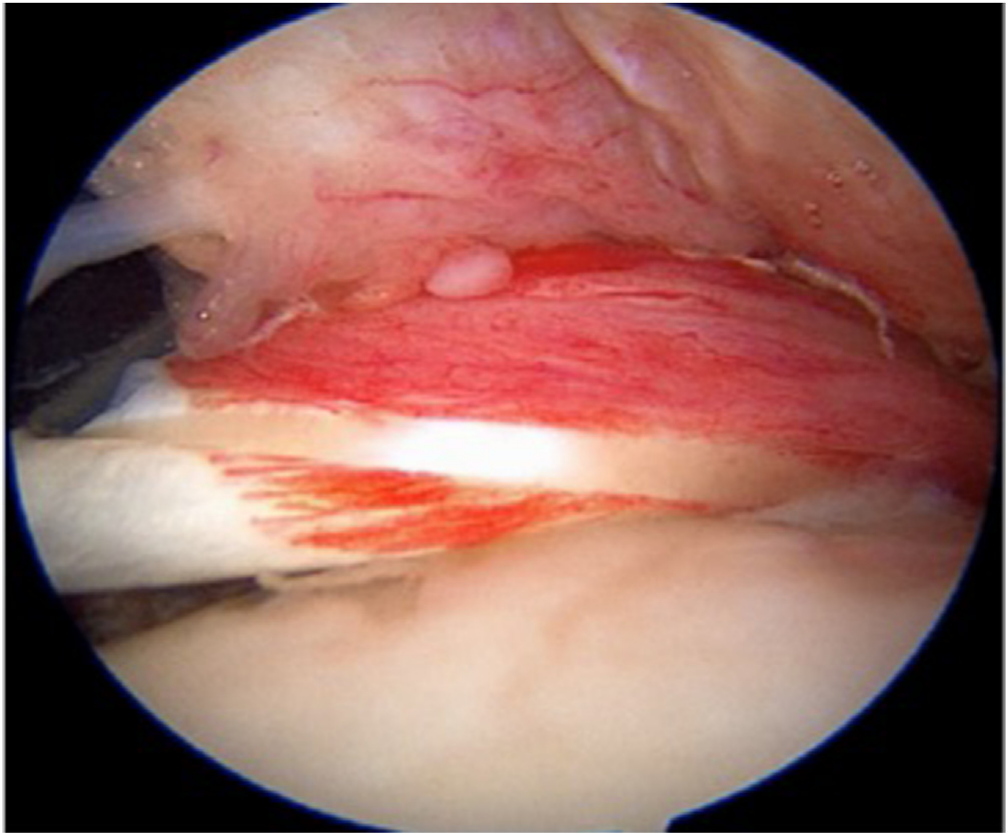
Arthroscopic image demonstrating a patient who exhibited severe tendonitis for which a tenodesis was performed. Viewing anterior from the posterior portal on a right shoulder.

Following Institutional Review Board approval, patients who underwent shoulder arthroscopy by a single surgeon (K.F.B.) between 2015 and 2018 for rotator cuff pathology, debridement, and distal clavicular excision (DCE), with or without subacromial decompression (SAD), and where the biceps tendon was left alone were retrospectively identified. Inclusion criteria were mild LS (defined as a biceps tendon with less than 50% of its surface area and that was hyperemic in appearance by arthroscopic examination), age between 18 and 89 years at the time of surgery, and a minimum two-year follow-up. The exclusion criteria were patients with any structural tearing or instability of the LHB, unstable significant superior labrum anterior-posterior (SLAP) tears, diagnosis of adhesive capsulitis, treatment with a tenodesis, or less than a 2-year follow-up. We classified the LS into mild or moderate to severe when assessing the biceps intraoperatively. Examples of tendons from this cohort that were considered mild and not surgically addressed during arthroscopy are seen in Fig 1, A-E. An example of what was considered moderate to severe LS (more than 50% of the biceps tendon with a hyperemic appearance by arthroscopy and addressed with a tenodesis) is shown in Fig 2.

Potential participants were mailed a letter or called to explain the purpose of the study, PROMs, and a return envelope. PROMs included American Shoulder and Elbow Surgeons standardized shoulder Assessment (ASES), simple shoulder test (SST), pain visual analog scale (VAS), and three site-specific questionnaires addressing surgical expectations. Once patients returned their consent form and PROMs, electronic medical records were used to obtain general demographics, comorbidities, concomitant surgical procedures, physical examination information from preoperative and postoperative clinic visits, and clinical outcomes. Data and PROMs were recorded on a data collection tool and scored.

The patients were separated into two groups. Group 1 consisted of those who reported bicipital groove tenderness presurgery, and Group 2 reported no bicipital groove tenderness presurgery. These two groups created the independent variables. The dependent variables were the standardized PROMs of the ASES, self-reported outcomes of the SST, and results from the VAS pain scale and site-specific questions regarding surgical expectations. Further data collected included demographic, baseline variables, and surgical outcomes.

SPSS Statistics for Windows, version 27.0 (SPSS Inc, Chicago, IL) was used to perform a multivariate analysis of variance (MANOVA) to analyze descriptive statistics and determine the main effect of bicipital groove pain on parametric outcome measures. The *P* value for significance was set to <.05. The site-specific questionnaire, which contained "Yes/ No" questions, required nonparametric testing using the Kruskal-Wallis test with the *P* value for significance set to <.017. Because of the nature of the nonparametric tests, the *P* value was set at <.017 to show no significant differences between the groups for their responses.

## Results

A total of 69 subjects were identified and sent the questionnaire. Sixty-four subjects responded (93% response rate). There were 36 subjects who experienced preoperative bicipital groove tenderness and 28 subjects with no preoperative bicipital groove tenderness. Demographic data are summarized in Table 1.

**Table 1:** Patient Demographics (*n*)

Parameter	Value
Number of shoulders	64
Mean age	54.4 (range 30 to 75 years)
Operative shoulder, right/left	50.0% (32) /50.0% (32)
Sex, male/female	48.4% (31)/51.7% (33)
Race	84.4% White (54), 7.8% Black (5), 1.6% Asian (1), or 6.3% Other (4)

**Fig 3:**
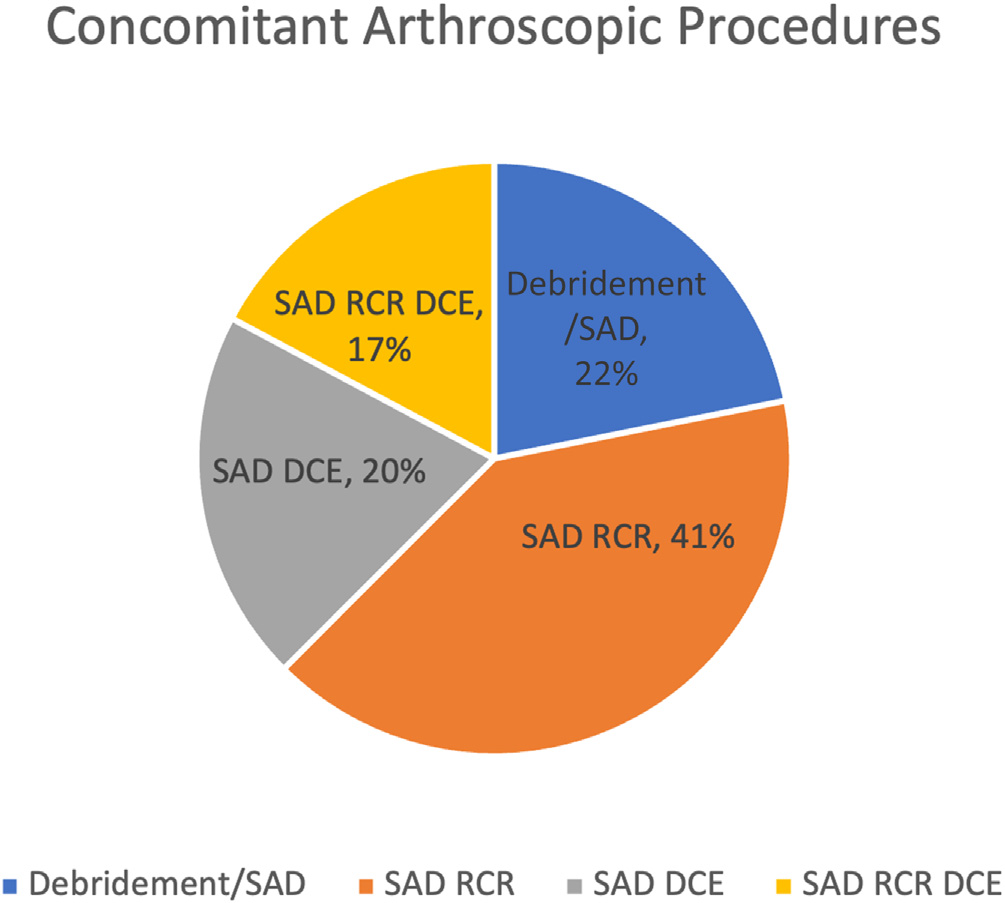
Distribution of concomitant arthroscopic procedures visualized by a pie chart with 22% (blue) of patients undergoing a debridement/subacromial decompression (SAD): 41% (orange) who underwent SAD and rotator cuff repair (RCR); 20% (gray) of patients who underwent SAD and distal clavicular excision (DCE); and 17% (yellow) of patients who underwent SAD, DCE and RCR.

All patients had either rotator cuff repair (RCR), RC debridement with subacromial decompression (SAD), distal clavicle excision (DCE), or a combination of the 3 procedures. The most common procedures done in sequential order from greatest to least were SAD and RCR (41%), debridement/SAD for impingement syndrome/bursitis (22%), SAD and DCE (20%), and SAD with RCR and DCE (17%). A breakdown of the number of patients receiving each procedure, the number of patients in each group that exhibited bicipital groove tenderness in each group, and the postoperative satisfaction in each group can be found in Table 2. Figure 3 demonstrates the specific distribution of concomitant arthroscopic procedures performed in the study.

**Table 2:** Types of Procedures Performed, Presence of Preoperative Bicipital Groove Tenderness, and Postoperative Satisfaction

	Bicipital Groove Tenderness	Patients (*n*)	Postoperative Satisfaction (Means ± SD)	*P* Value
SAD/Debridement Only	+	5	1.2 ± 2.17	.43
	−	9	0.5 ± 0.93	
SAD/RCR	+	11	0.1 ± 0.32	.33
	−	15	0.67 ± 1.76	
SAD/DCE	+	6	1.33 ± 2.07	.16
	−	7	0.14 ± 0.38	
SAD/RCR/DCE	+	6	0 ± 0	.30
	−	5	0.8 ± 1.79	

DCE, distal clavicle excision; RCR, rotator cuff repair; SAD, subacromial decompression.

There was no significant effect of whether a patient experienced bicipital groove pain or on PROMs: *V* = 0.04, *F*(6,55) = 0.49; *P* = .87. For the ASES scores, there was no significant difference in the scores of participants who had no bicipital groove pain (93.70 ± 11.83) and those who did (92.00 ± 15.31; *P* = .62). The SST scores showed no significant difference between those with no bicipital groove tenderness (10.66 ± 2.45) and those with groove tenderness (10.78 ± 1.87; *P* = .83). Patients with no tenderness (0.71 ± 1.74) and those with tenderness (0.93 ± 1.92) showed no significant differences for the subjective 0-10 VAS pain scale (*P* = .65). Similarly, patients with no tenderness (9.51 ± 1.27) and patients with tenderness (9.33 ± 1.75) demonstrated no significant difference in patient expectation scores (*P* = .64), with 10 being the highest level of postoperative expectation and 0 being the least. Lastly, patients reported their satisfaction on a scale of 0-10, whereas 10 indicated the most satisfaction with the procedure and 0 was the least. Patients with no tenderness (9.46 ± 1.38) and those with tenderness (9.44 ± 1.40) showed no significant difference in satisfaction scores (*P* = .92). All outcome measures had a low effect size of <.005. Table 3 compares the surgical outcomes between patients with preoperative biceps groove tenderness and those without tenderness.

**Table 3:** Comparison of Postoperative Scores Based on Preoperative Bicipital Groove Tenderness

	No Bicipital Groove Tenderness (*n* = 28)	Bicipital Groove Tenderness (*n* = 36)	*P* Value	Effect Size
	Means ± SD	Means ± SD		
ASES	93.70 ± 11.84	92.00 ± 15.31	.62	0.004
SST	10.66 ± 2.47	10.78 ± 1.87	.83	0.001
Pain	0.71 ± 1.74	0.93 ± 1.92	.65	0.003
Satisfaction	9.46 ± 1.38	9.44 ± 1.40	.97	0.000

ASES, American Shoulder and Elbow Surgeons Score; SD, standard deviation; SST, simple shoulder test.

For all nonparametric data, to analyze the site-specific “Yes/No” questions, a Kruskal-Wallis test was completed. In regard to needing additional surgeries, there was no significant difference between Groups 1 and 2; *P* = .38. Only 1 of the 64 subjects enrolled required additional surgery to address persistent anterior shoulder pain felt to be potentially related to her biceps. Following injections, which seemed to provide temporary relief, a revision arthroscopy and biceps tenodesis were performed. However, the patient's anterior shoulder pain persisted at the latest follow-up.

There was no significant difference between Groups 1 and 2 for the responses of perceived recovery (*P* = .96; 57/64; 89%) of subjects enrolled perceived making a full recovery after surgical intervention. Additionally, there was no significant difference between Groups 1 and 2 regarding whether the patient would undergo surgery again (*P* = .86; 62/64; 97%) of the subjects enrolled stated that if they could go back in time, they would choose to have the same procedure.

## Discussion

The most important finding of this study was that patients in whom the biceps was left alone in the setting of a mildly positive lipstick sign had a high patient-reported outcome scores with no significant differences between those who had preoperative groove pain and those who did not. Only 1 out of 64 patients underwent revision biceps tenodesis for persistent anterior shoulder pain, and this patient’s pain did not resolve following tenodesis. The results of this study showed that 97% of patients said they would have the surgery again. It is essential to point out that these patients’ indication for surgery was to address other, what was felt to be the more substantial shoulder pathology, both preoperatively and intraoperatively. This study was done to determine whether the right decision was made in cases where the surgeon was "on the fence" about whether the patient would benefit from a tenodesis. Additionally, it is important to note that the same surgeon who chose “benign neglect” for these patients performs ∼150 biceps tenodesis per year. Therefore, the cohort represented in the focus of this report is a relatively small percentage of patients undergoing shoulder arthroscopy.

When intraoperatively assessing a biceps tendon that has no structural damage or instability, there may be substantial interobserver variability among surgeons.^2^ Grassbaugh et al. found there is a relatively strong agreement in interobserver reliability between surgeons and detecting LHB erythema, with Cohen’s κ score range of 0.49 and 0.84 with most scores above 0.61.^2^ Intraobserver Cohen’s κ scores were generally above the suitable threshold of 0.6 for identifying and agreeing on biceps erythema and tendinopathy, with all values >0.6 for surgeons’ nominal (yes or no) assessment of erythema.^2^ In our study, we assessed the biceps by pulling it into the joint with the elbow flexed. The intraoperative grading of mild tenosynovitis or LS was judged by the surgeon. Grading was done via arthroscopic visual inspection of the long head biceps of the tendon. For the purposes of this study, tendons with less than 50% hyperemic surface area, not including the portion directly contacting the bicipital groove, were considered “mild.” Moderate to severe LS were cases with >50% hyperemic surface area of the biceps tendon; therefore, moderate/severe LS cases were not included in the study, as the biceps would have been addressed. Clearly, there may be variation in LS grading, as well as differences in surgeon thresholds to perform a tenodesis or tenotomy. Additionally, intraoperative examination of the biceps can miss more distal pathology, which is a possible cause of postoperative shoulder pain.^10^

**Fig 4:**
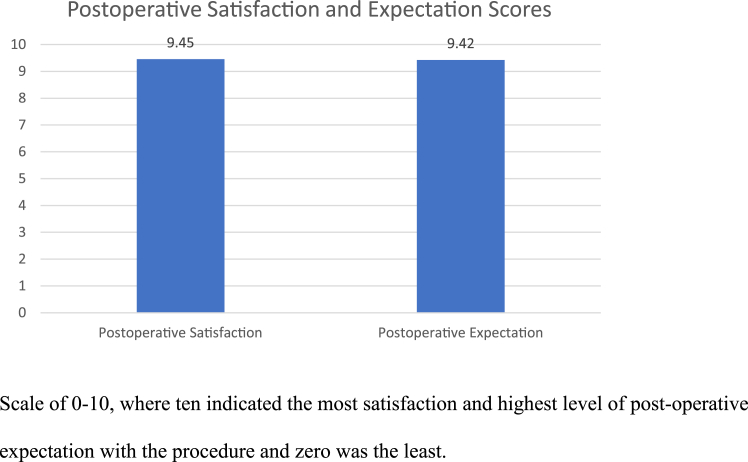
A histogram chart that compares patient postoperative satisfaction (left bar [9.45 out of 10]) and expectation scores (right bar [9.42 out of 10]), with 10 indicating no pain.

Some may consider anything other than a completely normal white tendon an indication to perform a biceps procedure. Ultimately, clinical decision-making in this setting is nonalgorithmic because of the subjective assessment of biceps pathology intraoperatively. Understandably, surgeons want to eliminate any possible pain generator for the patient, minimizing the chance of reoperation and optimizing outcomes. The principal finding of our results suggests that favorable outcomes can be found without tenodesis in patients with a mildly positive LS when the primary shoulder pathology is addressed. Furthermore, bicipital groove pain is nonspecific, and patients with impingement or bursitis can often have anterior shoulder pain; thus, groove pain should probably be termed anterior shoulder pain. The results of our study show that subjects who were the focus of this study were satisfied with their results, and 97% said they would elect to have their surgery again knowing in hindsight their ultimate result (Fig 4). The results support that the concomitant shoulder pathology that was addressed in this group was likely their primary pain generator. All but 1/64 (1.6%) subjects enrolled required no additional shoulder surgery, with the 1 patient having a complicated rheumatologic history, still finding no relief following biceps tenodesis, as mentioned earlier. In many of these patients, their postoperation rehab would have been prolonged significantly if a tenodesis were performed. A majority of these patients underwent rotator cuff debridement and SAD versus actual RCR, which is not reflective of the author’s practice. Despite the excellent patient-reported outcomes, one could argue that perhaps their pain and outcomes would have been better had the biceps been addressed.

Our results showed no correlation between preoperative bicipital groove tenderness and patient-reported outcome measures. One could argue that a tenodesis should be done anytime there is bicipital groove tenderness if the arthroscopic biceps exam reveals any pathology.^11^ Biceps tendon pathology is appropriately high on the surgeon’s differential diagnosis, especially when preoperative bicipital groove pain is present.^2^^,^^12^^,^^13^ However, anterior shoulder pain in the region of the bicipital grove is common and does not always correlate to biceps pathology.^14^ Several underlying shoulder disorders may cause anterior shoulder pain, and bicipital groove pain on exam can be nonspecific in terms of underlying etiology, especially in the setting of concomitant rotator cuff pathology.^14^ Although biceps injections can be helpful for diagnostic and therapeutic purposes, they are often not performed preoperatively if the biceps is not felt to be the primary pain generator or indication for surgery. Additionally, a recent study showed that ultrasound-guided bicipital groove injections might not be specific to alleviating biceps pathology, as the injection extravasates within the glenohumeral joint.^12^ Thus, pain relief from an injection may occur by treating other potential causes of anterior shoulder pain, including subacromial bursitis and rotator cuff-related pain.^1^ Persistent groove pain following tenodesis used to be faulted to the suprapectoral technique. However, there is also increased awareness that biceps tenodesis, even when performed in the subpectoral region, does not always successfully resolve anterior shoulder pain that is often described as bicipital groove pain.^10^^,^^15^ Recent systematic reviews have shown that patients undergoing arthroscopic biceps tenodesis (ABT) compared with open subpectoral biceps tenodesis (OBT) show no significant differences in postoperative “bicipital groove pain” between these 2 techniques.^15^ Thus, persistent anterior shoulder pain, which we felt to be groove pain, is often more complex and multifactorial than just residual biceps tendon pathology within the groove.

Preoperative bicipital groove pain or what we should probably term anterior shoulder pain in this group did not affect ultimate outcomes, which confirms sentiments that groove pain is not always helpful in the setting of concomitant shoulder pathology, such as rotator cuff tears. In this study, both ASES (93.70 ± 11.84 vs 92.00 ± 15.31; *P* = .62), and SST scores (10.78 ± 1.87 vs 10.66 ± 2.47; *P* = .83) were comparable no matter if they had preoperative “groove pain” or not. Patients with groove tenderness (0.93 ± 1.92) and no tenderness (0.71 ± 1.74) also showed no significant difference for the subjective 0-10 VAS pain scale (*P* = .65). Although evaluation for anterior shoulder pain or groove pain is important in the preoperative evaluation, our results indicate that it may be reasonable to leave the biceps alone in the setting of mild inflammation whether the patient has preoperative bicipital groove tenderness or not. However, this ultimate decision-making by the surgeon should be done in the context of other underlying pathology. Although we did not evaluate on exam at 2 years postoperation if the subjects enrolled had postoperative bicipital groove tenderness after addressing their primary pathology, our results revealed that their pain levels were reported to be quite good, and patients were quite satisfied with their outcomes. Understandably, however, one could still argue that pain levels could still be improved, and they perhaps could have been better if a tenodesis had been performed.

Some studies reveal the LS may only be 49% sensitive and 67% specific for detecting LHB tendinitis and just 64% sensitive and 32% specific for identifying true pathology by detecting erythema about the LHB tendon.^2^ There is currently no grading scale for biceps tenosynovitis or the LS, which is a potential future area of investigation. Additionally, there can be variability in inflammation in the tendon depending on when it is evaluated during the arthroscopy. Especially when epinephrine is used in the arthroscopy solution, the inflamed biceps can look better and better, as it is exposed to the irrigation fluid. It is our habit to evaluate the biceps tendon at the initiation of the diagnostic arthroscopy since this is likely going to reflect the inflammation appreciated visually, most accurately. This may not be the case with all surgeons, which can also lead to interobserver variability in assessment of the tendon and decision-making.

For many surgeons, intraoperative decision-making related to indications for performing biceps surgery has evolved. The era of debridement or nonoperative treatment, unless 50% of the tendon was torn, severely inflamed, or unstable, has given to an age of many self-proclaimed "biceps killers.”^7^^,^^14^ Of note, during the 3 years when these patients were collected, the same surgeon performed over 400 biceps tenodesis. It is interesting to wonder if the pendulum has swung too far. Are we okay to leave the biceps alone in the setting of mild tenosynovitis (i.e., LS), especially if it is felt that other concomitant shoulder pathology is the patient's problem? Does leaving a mild LS alone place the patient at risk of subsequent biceps tenodesis or inferior postoperative outcomes? Most of us now have a much lower threshold than 20 years ago for treating the biceps with a tenodesis or tenotomy. Nobody currently refutes addressing the biceps in the setting of more severe tenosynovitis. The purpose of this study is not to advocate for leaving the biceps alone in the setting of a mild LS, but to justify the surgeon doing so if they feel benign neglect is in the patient's best interest.

Tenodesis is associated with possible adverse events that must be weighed against the undesirable risk of revision and inferior outcomes if a pain-generating pathologic biceps tendon is left alone.^9^^,^^14^ If there were no downsides to addressing the biceps, the consensus should be to have a very low threshold to sacrifice the tendon unless it is normal during concomitant procedures. Fortunately, most patients do well following tenodesis or tenotomy^9^^,^^10^^,^^16^; however, complications can occur.^9^ Many surgeons prefer tenodesis over tenotomy to preserve the bicep’s length-tension relationship, maintain normal anatomy, and minimize power loss.^17^ However, potential adverse events of tenodesis are known, including loss of fixation/failure and subsequent "Popeye sign," length-tension mismatch, persistent biceps pain, shoulder stiffness, infection, hardware-related problems, hematoma, fracture, neurologic injuries, vascular injuries, and reflex sympathetic dystrophy.^9^ Although variable, some report failure rates at 20% when tenodesis is performed with interference screw fixation.^16^ Other studies report that 20% to 49% of patients may demonstrate residual bicipital groove tenderness after tenodesis.^17^^,^^18^

Several studies and anecdotal estimates report that surgeons are performing an increasing number of tenodesis procedures relative to 10-20 years ago.^19^, ^20^, ^21^, ^22^ In one meta-analysis, authors found that 77% of patients treated with biceps tenodesis, and 89% with biceps tenotomy, had a concomitant RCR.^23^ Some surgeons have self-admittedly become more aggressive regarding their intervention threshold for biceps tenodesis.^18^ Surgically addressing the biceps is often justified, and the authors are currently doing biceps tenodesis in a large percentage of their rotator cuff repair patients. The question is, have we lowered our thresholds to operate on the biceps too far? Were rotator cuff outcomes compromised 10-30 years ago due to not addressing the biceps often enough? Reoperation for failure to address the biceps was not very common.^24^ Most surgeons practicing during that era or published studies do not report high failure rates due to not adequately addressing the biceps based on previous strategies.^7^ Clearly, we can appreciate and diagnose biceps pathology much better with our current arthroscopic techniques than with older open approaches. It makes sense to address pathology if it improves patient outcomes. We just need to always remember we can sometimes overtreat and that doing something does not always impart better results.

Finally, this study highlights the need to differentiate the severity of biceps inflammation and the lipstick sign to facilitate communication and intraoperative decision making. Currently, most surgeons agree that in the setting of a markedly erythematous, unstable, or structurally damaged LHB requires intervention for optimal outcomes. We should stress we are reporting on a group where the biceps inflammation or erythema (LS) was assessed to be mild. There is little consensus on when to address the biceps when the inflammatory pathology is mild. A potentially limiting factor to extrapolating these results is that different surgeons may still have varied subjective opinions on what constitutes a mild LS that may not require intervention.

### Limitations

Our study has several limitations. There is little consensus regarding the clinical distinction between grades of pathology when it comes to the lipstick sign. Therefore, interobserver and intraobserver reliability may not be consistent. Surgeon bias may have been present when deciding to leave the biceps alone, as many other patients received tenodesis throughout the study period. Additionally, assessing the biceps in the groove with the elbow flexed does not assess more distal biceps pathology, which, if missed, may negatively influence postoperative pain scores and PROMs.

## Conclusion

Patients with mild biceps tendonitis showed favorable outcomes with low revision rates and high patient satisfaction when the biceps tendon was not surgically addressed when the primary shoulder pathology was treated during arthroscopy. Outcomes were not affected by the presence of preoperative groove pain.

## Supplementary Data

ICMJE author disclosure forms
